# What Do We Know about Individuals Who Are Assessed as Being at Moderate Risk for Future Fracture in Canada?

**DOI:** 10.4236/health.2015.75061

**Published:** 2015-05

**Authors:** Joanna E. M. Sale, Ravi Jain, Kosalan Akilan, Kevin Senior, Dorcas Beaton, Earl Bogoch, Gilles Boire, Marie-Claude Beaulieu, David Lightfoot, Larry Funnell

**Affiliations:** 1 Musculoskeletal Health and Outcomes Research, Li Ka Shing Knowledge Institute, St. Michael’s Hospital, Toronto, Canada; 2 Institute of Health Policy, Management & Evaluation, University of Toronto, Toronto, Canada; 3 Osteoporosis Canada, Toronto, Canada; 4 Mobility Program, Li Ka Shing Knowledge Institute, St. Michael’s Hospital, Toronto, Canada; 5 Department of Surgery, University of Toronto, Toronto, Canada; 6 Department of Medicine, Division of Rheumatology, Université de Sherbrooke, Sherbrooke, Canada; 7 Department of Family Medicine and Emergency Medicine, Université de Sherbrooke, Sherbrooke, Canada

**Keywords:** Moderate Risk, Osteoporosis, Management, Outcomes, Scoping Review

## Abstract

**Objective:**

We examined what was known about individuals in Canada who were assessed as being at moderate risk for future fracture.

**Methods:**

A scoping review was conducted. Eligible articles were Canadian studies published from 2010 onwards reporting on primary data that included patients at moderate risk for future fracture. We limited the search to Canada as fracture risk categorization is unique to each country. Studies were identified by searching relevant databases. Two reviewers independently reviewed titles and abstracts to determine each study’s eligibility. General information about each study, demographic information about the moderate risk groups (including tool used to determine moderate risk (Fracture Risk Assessment Tool (FRAX), Canadian Association of Radiologists and Osteoporosis Canada (CAROC)), and outcomes (number of patients: recommended treatment, prescribed treatment, initiating treatment, persisting with treatment after six months, who re-fractured, who died) were documented.

**Results:**

We identified 1193 papers which were further screened for eligibility. Of the 1193 identified, 7 were eligible for the review but only 4 articles contained demographic or outcome data on moderate risk patients. In one study, 1.8% of moderate risk patients died over a mean 5.3 years of observation and in three studies, the risk of fracture was 5.9% over a median of 3 years of follow-up, 8.3% over a mean of 5.4 years, and 14.7% over 10 years of follow-up.

**Conclusion:**

There is a wide knowledge gap in the literature concerning individuals who are assessed as moderate risk for future fracture in Canada.

## 1. Introduction

Recent years have seen a shift in treatment guidelines from a *diagnosis of osteoporosis* (*OP*) to *fracture risk*. In the United Kingdom, the National Osteoporosis Guideline Group recommends that fracture risk is calculated first using the Fracture Risk Assessment Tool (FRAX) without bone mineral density (BMD) to categorize low, intermediate or high risk probabilities for fractures at ten years [1] [2]. FRAX with BMD is then used to further classify patients with intermediate risk to the low or high risk group; treatment guidance does not apply to intermediate risk patients. In Canada, the 2010 Clinical Practice Guidelines for the Diagnosis and Management of OP [3] recommend that patients are assessed by considering a number of clinical factors and then using either FRAX [4] or the revised tool by the Canadian Association of Radiologists and Osteoporosis Canada (CAROC) [5] to determine fracture risk. Patients at high risk for future fracture (>20% ten year risk) are recommended pharmacotherapy and patients who are at low risk (<10% ten year risk) are not recommended pharmacotherapy. However, recommendations for patients at moderate risk are vague, regardless of the patient’s history of fragility fracture (occurring after a slip, trip, or fall from standing height or less [6]). Based on unpublished data, approximately 61% of participants screened through the Fracture Clinic Screening Program in Ontario are moderate risk as determined by the 2010 Canadian guidelines (CAROC). Thus, management of the majority of fragility fracture patients in Canada relies primarily on clinical judgment, rather than evidence.

We conducted a scoping review [7] to summarize what was known (demographics, management, outcomes) about individuals who were at moderate risk for future fracture in Canada. Specifically, our objectives were to: a) examine demographic characteristics, management, and outcomes for individuals at moderate risk for future fracture; and b) identify research gaps in the existing literature regarding these individuals.

## 2. Materials and Methods

A scoping review [7] was under taken from July to September 2014. Our team was comprised of clinical epidemiologists (JS, DB), knowledge users (RJ, LF), an Information Specialist (DL), clinicians (EB, DB, GB, M-CB), a consumer representative (LF), and two undergraduate students (KA, KS).

### 2.1. Identifying Relevant Studies

Eligible articles were those that: 1) were Canadian studies reporting on primary data; 2) included patients at moderate risk for future fracture; and 3) were published from October 2010 onwards. We limited the search to Canada as fracture risk categorization is unique to each country and clinical practice guidelines on how to manage fracture risk are heterogeneous worldwide. We also limited the search to 2010 onwards as this is the year the most recent Clinical Practice Guidelines for the Diagnosis and Management of OP in Canada were published [3].

Using Arksey and O’Malley’s approach [7], we identified relevant studies written in English or French by searching the databases MEDLINE, CINAHL, Cochrane Database of Systematic Reviews, EMBASE, PsycIN-FO, Social Sciences Abstracts, and Scopus. The Information Specialist worked with the authors to develop and refine the search strategy. A general list of the descriptors for the search domains included: 1) “fracture” or “osteoporosis” or “bone”; 2) “fracture risk”; and 3) “Canada”, or terms for the individual provinces and territories. The Information Specialist searched each database independently, combined the results into a single Reference Manager database, removed the duplicates, and exported the results into an Excel spreadsheet.

### 2.2. Study Selection

Two reviewers (KS, KA) independently reviewed titles and abstracts to determine each study’s eligibility, as recommended [8]. If the eligibility of the article was not clear from the title and/or abstract, the full article was retrieved to determine if the article met the inclusion criteria. If articles failed to meet one eligibility criterion, they were not reviewed further. Questions about individual articles and disagreements between the two reviewers were discussed with the first author who made a final decision on whether an article was eligible or not. As recommended [9], all studies that were deemed eligible for the review were retrieved, assigned a unique identifying number, and downloaded into a shared folder.

### 2.3. Charting the Data

The two reviewers and the first author read the full text of the eligible papers and under the first author’s supervision, the two reviewers independently charted general information about each study (authors, year of publication, title, study location, sample size), demographic information about the moderate risk groups (age, sex, patients presenting with a fracture (Y/N), patients screened through a post-fracture secondary prevention program (Y/N), comparison group (high risk, low risk, other)), tool used to determine moderate risk (FRAX with or without BMD; CAROC), and outcomes (see below). As recommended [8], the two reviewers and the first author met after data extraction from the first 10 studies to ensure consistency of the charting approach with the research question and purpose. Procedures and steps were similar to those followed for systematic reviews [10]. We used PICOS [11] as a framework for our charting and documented the following elements.

#### 2.3.1. Population

We documented whether patients presented with a fragility fracture or not: “Fracture patient—Yes/No”.

#### 2.3.2. Intervention

We did not focus on any particular type of intervention but reported which patients were screened through a post-fracture secondary prevention program as described [12]–[15] versus those who had not been screened through such a program. This was documented as: “Post-fracture intervention—Yes/No”.

#### 2.3.3. Comparison Group

Our comparison groups were patients at “low risk for future fracture”, patients at “high risk for future fracture”, and “Other”. This was documented as: “Comparison group—Low risk, High risk, or Other”.

#### 2.3.4. Outcomes

We included both short-term and long-term outcomes as identified in our team’s previous systematic review [12] [16] [17]. Short-term outcomes included the number of patients recommended treatment, prescribed treatment, and initiating treatment. Long-term outcomes included number of patients persisting with treatment after six months, who fractured, and who died. We classified “treatment” as “pharmacotherapy”, “supplement use”, and “other”. “Other” treatment referred to exercise training, falls reduction programs, and the use of aids and mobility devices.

#### 2.3.5. Study Design

We identified all literature on moderate risk patients regardless of study design [7]. The design of each study was documented as: “Randomized controlled trial”, “Cohort (prospective, retrospective)”, “Cross-sectional”, “Case-controlled/Case series”, “Chart review”, “Qualitative”; or “Other”.

## 3. Results

**Figure 1:**
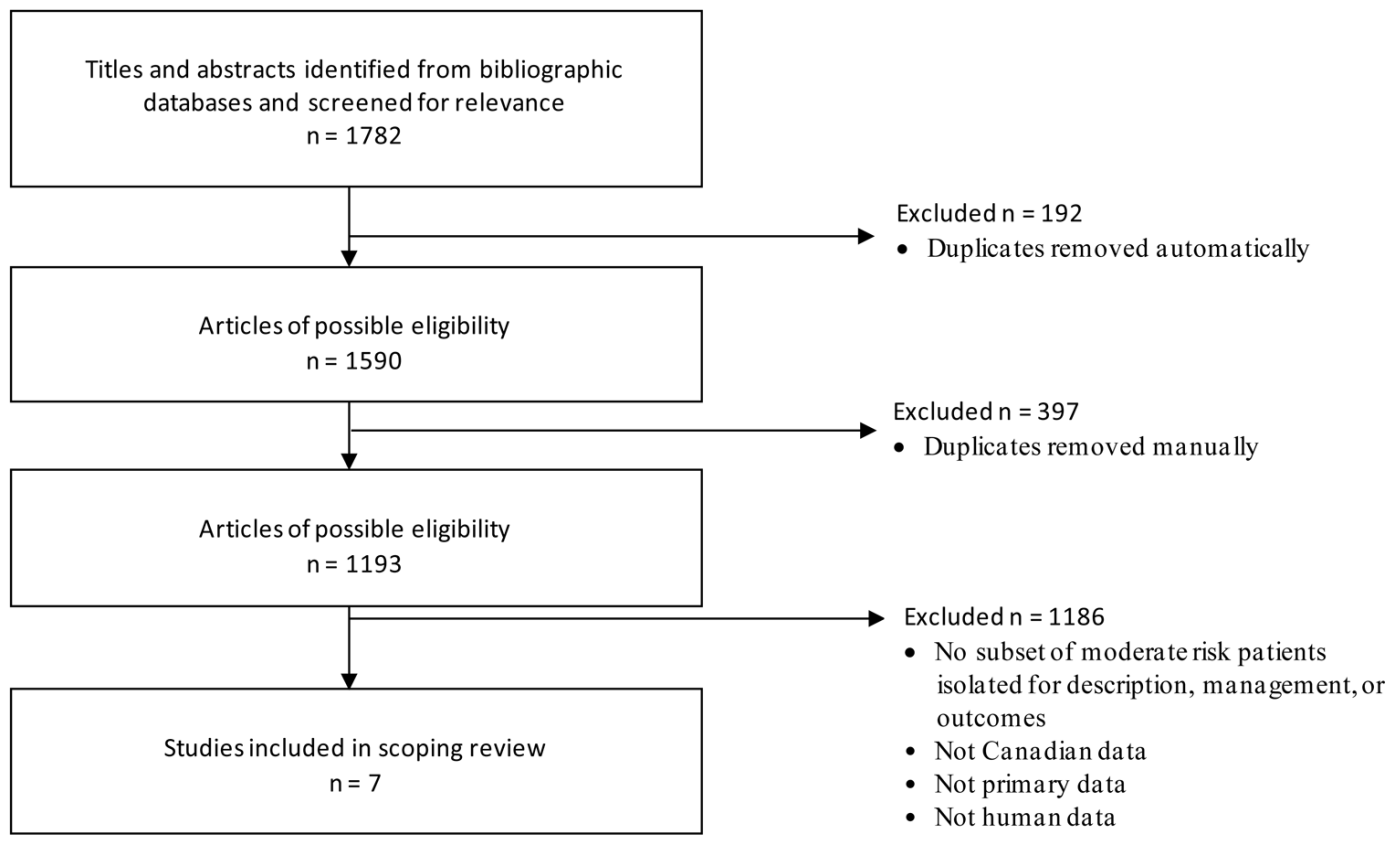
Results of literature search to identify studies on moderate risk patients.

We identified 1782 papers which were further screened for eligibility. All articles were written in English. Duplicates were removed automatically by the software program and then manually, resulting in 1193 articles (see Figure 1). Fifty-six citations were abstracts that we followed up to retrieve the full paper either by searching the internet (n = 51) or by contacting the first author of the abstract (n = 5). We also contacted the authors of two additional articles identifying a moderate risk group but no demographic or outcome data on this group. Of the 1193 identified, seven [18]–[24] were eligible for the scoping review (see Table 1).

Of the seven articles, one [23] focused on patients recruited from a post-fracture secondary prevention program while the remaining articles included patients with and without a fracture. Four of the seven articles were based on data from the province of Manitoba. Most of the studies relied on FRAX to determine fracture risk assessment. One study [23] used FRAX without BMD to determine fracture risk but the moderate risk group data were generated from a CAROC calculation in a subset of patients with available BMD; the FRAX scores were categorized as low-moderate versus high. Three of the seven articles [20] [21] [24] relied on a previous version (CAROC 2005) of the fracture risk tool referenced in the 2010 Canadian guidelines. Two of these papers [20] [21] were retained as CAROC 2005 was used only for categories as low (<10%), moderate (≥10% to ≤20%), and high (>20%) and 10-year fracture risk estimation was based on FRAX. Of the six articles using the fracture risk tool from the 2010 guidelines, the only demographic data reported on the moderate risk group was a mean age of 71.4 years in the Manitoba database [21]. Four studies reported outcome data on the moderate risk group [19]–[21] [23]. Roux and colleagues [23] reported that 5.9% of moderate risk patients from a post-fracture cohort in Quebec re-fractured over a median of 3 years of follow-up (see Table 2). In two studies, the observed incidence of fractures in FRAX with BMD-defined moderate risk patients from the Manitoba Bone Mineral Density database was 8.3% with a mean of 5.4 years of observation [21] and, in a subset of patients, 14.7% over 10 years [20]. In one Manitoba study [19], 1.8% of moderate risk patients died over a mean 5.3 years of observation. No other short- or long-term outcomes were reported.

## 4. Discussion

We identified a knowledge gap in what is known about individuals who are moderate risk for future fracture in Canada. Few studies identified in our scoping review used the 2010 guidelines. A Manitoba cohort represented 4 of the 7 studies that were eligible and only one study included a post-fracture intervention [23]. The majority of studies examined validity and measurement properties of fracture risk assessment tools, for example, implications of minor adjustments to the FRAX tool. However, few studies stratified their samples to isolate and describe the moderate risk group. In other countries, such as the UK, a similar category labeled the “intermediate” group is further categorized to a low or high risk category using FRAX with BMD [1] [2], which may partly explain the lack of information on the management of the intermediate, or equivalent, group worldwide.

Most studies relied on FRAX for fracture risk assessment. In Canada, FRAX scores [4] are often mapped to high/moderate/low categories derived from CAROC [5]. Although the Canadian guidelines recommend both CAROC and FRAX [3] [25], the appeal of using FRAX is that it has been validated internationally, can be used without BMD in many patients, and it accounts for the presence of one or more risk factors not accounted for by CAROC (e.g. parental history of hip fracture, smoking) [3] [26]. Clinical members of our research team have used CAROC more than FRAX which may imply there is a difference in preference of tools between researchers and clinicians.

There is widespread uncertainty about how to identify and manage patients who are assessed as moderate risk for future fracture, partly because there are no published data specifically addressing that subgroup. Wall and colleagues [27] examined fracture risk assessment in long-term care physicians in Ontario, Canada, and reported that only 54% of physicians correctly evaluated a patient to be at moderate risk. We believe it is especially important to have clearer guidelines for treating moderate patients who have sustained a fracture as unpublished data from our group (JS, RJ, DB, EB) show that the majority of patients who have sustained a fracture are assessed as moderate risk.

One long-term implication of our scoping review is that it will inform future clinical practice guideline development. The focus of the current Canadian guidelines is on the “highest risk” population and patients at moderate risk for future fracture have received little clinical focus or program planning. Thus, the individual clinician is left to decide whether or not to treat patients in the moderate group. We propose that future research needs to identify and study the outcomes of the moderate risk group including the possibility of re-evaluating their risk classification, especially in the context of post-fracture interventions.

In order for recommendations to be followed by physicians in real world settings, it is important to have a simple clear message about the guidelines; algorithms should fit on a single page. At the same time, a clear communication strategy needs to be developed to provide patients with a better understanding of what it means to be in the moderate risk category and how they can minimize their risk of future fractures.

## 5. Conclusion

In conclusion, there is a wide knowledge gap in the literature concerning individuals who are moderate risk for future fracture in Canada. One limitation of our study is that we conducted it four years after the 2010 guidelines were published so it may be premature to demonstrate uptake of the guidelines. However, our findings and recommendations are supported by a diverse team representing researchers, consumers, and clinicians, including an orthopaedic surgeon, specialists, and a family physician who regularly assess moderate risk patients.

## Figures and Tables

**Table 1:** Description of eligible studies.

Article ID	First author, year of publication	Study location	Age (mean or median, range)	Sex (M/F)	Tool used to determine moderate risk	Study design	Fracture patient (Y/N)†	Post-fracture intervention (Y/N)	Comparison group (low, high, other)	Sample size (including moderate risk subset)
36	Brennan, 2014 [18]	Manitoba	Mean 65.9, 50+	F	FRAX with and without BMD	Cohort	Y/N	N	Low, high	51,327
378*	Allin, 2013 [24]	Ontario	Mean 67.2, >40	M/F	CAROC	Cluster randomized trial	Y/N	N	Low, high	48
573‡	Leslie, 2011 [20]	Nine Canadian Cities**	Mean 65.6, 50+	M/F	FRAX with BMD	Cohort	Y/N	N	Low, high	6388
1035	Roux, 2014 [23]	Quebec	Median 67, 50+	M/F	CAROC; FRAX with and without	Cohort	Y	Y	High; other	1409
1883	Giangregorio, 2012 [22]	Manitoba	>50	M/F	BMD FRAX with BMD	Cohort	Y/N	N	Low, high	39603
1902‡	Leslie, 2012 [21]	Manitoba	50+	M/F	FRAX with and without BMD	Cohort	Y/N	N	Low, high	39603
1904	Leslie, 2013 [19]	Manitoba	50+	M/F	FRAX with BMD	Cohort	Y/N	N	Low, high	39603

* Used the 2005 CAROC for determining fracture risk;

** St. John’s, Halifax, Quebec City, Toronto, Hamilton, Kingston, Saskatoon, Calgary & Van-couver.

† Y/N denotes that some patients had previous fractures, some did not.

‡ Used the 2005 CAROC for the *categories* of fracture risk which were then determined using FRAX.

**Table 2:** Outcomes for moderate risk groups.

Article ID	First author, year of publication	Number (%) recommended treatment*	Number (%) prescribed treatment*	Number (%) initiating treatment*	Number (%) persisting with treatment > 6 months*	Number (%) who re-fractured	Number (%)who died
573	Leslie, 2011 [20]					217 (14.7)≠	
1902	Leslie, 2012 [21]					1021 (8.3)¥^b^	
1904	Leslie, 2013 [19]	-	-	-	-	-	220 (1.8)**
1035	Roux, 2014 [23]	-	-	-	-	9 (5.9)#	-

≠ Over 10 years of observation;

¥ Over a mean of 5.4 years of observation;

b FRAX with BMD; 909 (7.8%) fractured if used FRAX without BMD;

* Treatment refers to pharmacotherapy for bone health;

** Over a mean 5.3 years of observation;

# Over a median of 3 years of follow-up.
